# Modelling the alpha and beta diversity of copepods across tropical and subtropical Atlantic ecoregions

**DOI:** 10.1038/s44185-025-00073-x

**Published:** 2025-01-31

**Authors:** Lorena Martínez-Leiva, José M. Landeira, Maria Luz Fernández de Puelles, Santiago Hernández-León, Víctor M. Tuset, Effrosyni Fatira

**Affiliations:** 1 Instituto de Oceanografía y Cambio Global, IOCAG, Universidad de Las Palmas de Gran Canaria, Unidad Asociada ULPGC-CSIC, Campus de Taliarte, 35214 Telde, Gran Canaria, Canary Islands, Spain; 2 Department of Biology, Norwegian University of Science and Technology, Trondhjem Biological Station NO-7491 Trondheim, Trondheim, Norway; 3 Instituto Español de Oceanografía (IEO/CSIC). Centro Oceanográfico de Baleares (COB), Muelle de Poniente s/n, 07015 Palma, Spain

**Keywords:** Biodiversity, Biogeography, Biooceanography, Ecology, Ocean sciences

## Abstract

Copepods, the most abundant individuals of the mesozooplankton, play a pivotal role in marine food webs and carbon cycling. However, few studies have focused on their diversity and the environmental factors influencing it. The objective of the present study is to model the alpha and beta diversity of copepods across the tropical and subtropical ecoregions of Atlantic Ocean using both taxonomic and functional approaches. The study used a dataset of 226 copepod species collected by stratified plankton hauls (0–800 m depth) across the tropical and equatorial Atlantic, from oligotrophic waters close to the Brazilian coast to more productive waters close to the Mauritanian Upwelling. To perform the functional analysis, six traits related to the behaviour, growth, and reproduction of copepods were selected. Several alpha diversities were estimated using taxonomic metrics (*SR, Δ+, and Λ+*) and functional metrics (*FDis, FEve, FDiv, FOri, FSpe*), and modelized with GAM model across spatial and environmental gradients, and day/night. The overall and two components of *β*-diversity (turnover and nestedness) were shared between depth and stations. The surface layers of stations from oligotrophic, equatorial, and Cape Verde ecoregions displayed higher values of taxonomic *α*-diversity. More unpredictable were the facets of functional *α*-diversity, although they showed a tendency to be positive with depth during the daytime. The GAM analysis revealed spatial gradients as the key factors modelling the taxonomic *α*-diversity, whereas depth was the most relevant for functional *α*-diversity. The turnover component drove taxonomic *β*-diversity in depth and station, whereas the nestedness component acquired relevance for the functional *β*-diversity. The taxonomic structure of the copepod community varied spatially across depths and ecoregions, but this was not linked to functional changes of the same magnitude.

## Introduction

Understanding the dynamics of zooplankton communities is of significant scientific interest, as these organisms play a critical role in vertical energy flux in both marine and freshwater ecosystems^1^^,2^. Many studies have primarily focused on geographical and environmental factors influencing zooplankton abundance and distribution, often using ecological and taxonomic indices to describe diversity^3,4^. However, functional diversity, which involves understanding communities and ecosystems through the roles organisms perform, has transformed perspectives on biodiversity. It is now well-established that functional trait combinations in zooplankton are closely related to environmental factors^5–11^. In this context, examining the functional characteristics of communities at the local scale (α-diversity) is crucial, as it provides insight into species interactions with their environment, the maintenance of ecosystem processes, and community responses to environmental changes^8,12^. Additionally, functional *β*-diversity, which represents the dissimilarity in trait composition across spatial gradients, has emerged as another key component to consider in ecological studies^13–18^. *β-*diversity is partitioned into turnover and nestedness^19^. Turnover is the species replacement without changing species richness, while nestedness component accounts for variations in richness due to species gain or loss. These two components collectively shape the overall dissimilarity among communities, with their relative significance fluctuating based on the ecological processes governing community structure^20,21^. Understanding the contribution of each component is crucial for implementing conservation strategies aimed at preserving regional species diversity^22^. When the nestedness component dominates, indicating low complementarity among sites, it implies the necessity to prioritize sites with high *α-*diversity. Conversely, when the turnover component is predominant, conservation efforts should be directed towards multiple sites^12,23,24^. However, studies assessing the *β-*diversity of zooplankton in marine ecosystems remain largely unexplored, with research efforts predominantly focusing on estuaries, ponds, and rivers rather than marine environments (refs. ^25–29^).

Copepods, the most abundant individuals within the mesozooplankton group across the oceans^30,31^, play a central role in marine ecosystems. They serve as essential links in marine food webs connecting primary producers with higher trophic levels^32–34^ and contribute significantly to the biological carbon pump and global biogeochemical cycles^35,36^. Throughout diel vertical migrations (DVM), copepods graze on microplankton in the epipelagic layers and export organic matter (e.g. fecal pellets) below the eutrophic zone^36,37^. Indeed, the size of copepods is directly correlated with the size of fecal pellets, and therefore, the proportion of carbon exported^5,38^. Consequently, changes in the structure of the copepod community can affect carbon sequestration and the overall functioning of marine ecosystems^39^. Within this context, the abundance, size, distribution, and diversity of copepods are influenced by environmental factors such as temperature^40^, dissolved oxygen levels^41^, food availability^42^, and spatial gradients^30,43^. Biodiversity decreases poleward^5,44^ and with the depth^45,46^, exhibiting notable variations across different regions of the ocean^45^.

Feeding patterns extend throughout the water column, with carnivores and detritivore copepods increasing in the bathypelagic layers. According to the niche-based hypothesis, the environment acts as a filter that shapes the identity and abundance of species within a community based on their functional traits^47^. Coexisting species exhibit slight variations in their functional traits, as suggested by the niche filtering hypothesis^48^, and the local exclusion of species that are very similar in their resource requirements is known as limiting similarity^49^. For example, under strong resource competition conditions, the presence of ambush-feeding copepods increases because of their lower energy consumption^31^. This food limitation prompts copepods to adopt carnivorous strategies, including cannibalism^50^. Predominantly, most carnivores are large copepods with mixed or ambush strategies, requiring active swimming. Conversely, smaller copepods are passive feeders that consume less energy^11^. Feeding patterns are extend throughout the water column, with carnivores and detritivore copepods increasing in the bathypelagic layers^11^. Moreover, spawning strategies are influenced by oceanographic conditions, with sac-spawners prevailing in warm waters and broadcasters in colder waters^44^. Therefore, the distribution of copepod species depends on functional features that influence species fitness, encompassing key aspects of the ecology, physiology, morphology, and behavior^51–57^. Based on this, Benedetti et al^5^. described eleven different functional groups of copepods in a global-scale study.

The present study aims to assess copepod diversity and community structure along the tropical and equatorial Atlantic Ocean^58,59^. The *α-* and *β-*diversities were estimated and modelled at spatial and temporal (day/night) scales using taxonomic and functional metrics. The main objectives of this study were: (a) to determine the characteristics of functional groups present in the central Atlantic Ocean, (b) to quantify the variability of α- and *β*-diversity across taxonomic and functional facets, (c) to assess the contributions of different environmental drivers to these multifaceted components of α-diversity, and (d) to identify which component of *β*-diversity (turnover or nestedness) better explains changes at a spatial scale. We hypothesize that: 1) the facets of α- and β-diversity will vary along the environmental gradient in the central Atlantic, 2) stations near the African upwelling coast will exhibit higher species richness and likely lower functional impact due to an increase in the nestedness component of *β*-diversity, and 3) functional *β*-diversity will be low across all localities, given the limited number of functional groups identified by Benedetti et al.^5^ both globally and within our specific study region.

## Material and methods

### Sampling and study area

**Fig. 1:**
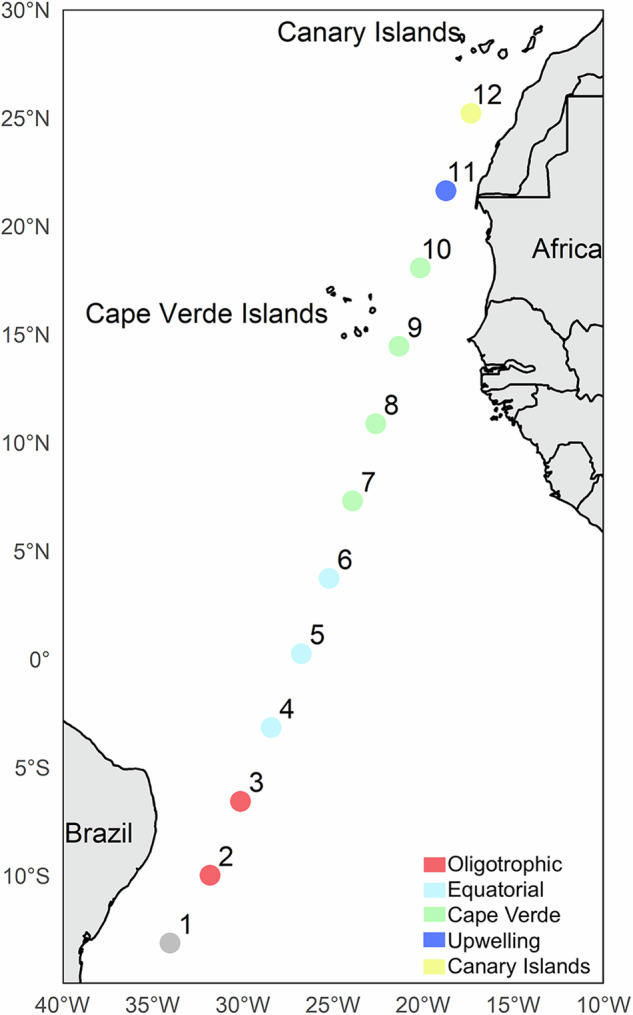
Location of stations sampled along the tropical and subtropical Atlantic Ocean. Colours according to ecoregions^58^. St#1 was not sampled.

A presence/absence dataset of copepods species, with 226 species^43^, was built using samples collected across the tropical and equatorial Atlantic on-board R/V Hesperides during “Migrants and Active Flux in the Atlantic Ocean (MAFIA)” cruise (in April 2015). The cruise took place in a latitudinal transect along 12 stations 420 km apart, from 500 km off the Brazilian coast to 200 km south of the Canary Islands (Fig. 1). At each station, except for the St#1, mesozooplankton samples were collected during day and night using a MOCNESS–1 net (Wiebe et al., 1985) fitted with 0.2 mm meshes. This multinet allows the collection of samples across seven different depth strata per haul: 800–600 m, 600–500 m, 500–400 m, 400–300 m, 300– 200 m, the lower thermocline layer (ca. 200–100 m), thermocline (ca. 50–100), and the upper mixed layer (ca. 50–0 m). Two Conductivity-Temperature-Depth (CTD) casts were performed at each station using a Seabird 911Plus instrument with a Seabird-43 Dissolved Oxygen Sensor and a Seapoint Chlorophyll Fluorometer Sensor. Thus, the environmental variables obtained were depth (m), fluorescence (volts), density (kg·m^−^^3^), oxygen (μmol·kg^−^^1^), temperature (°C), chlorophyll (mg·m^−^^3^), and salinity (PSU).

Following previous analysis of the environmental condition of this cruise, the tropical and equatorial Atlantic is divided into five regions^58^ (Fig. 1). In the western section of the transect we identified two regions. (1) The oligotrophic region (St#2-3), characterize by deep and low values of chlorophyll, and high temperature and salinity in the upper layers, that dropped significantly below 400 m depth. (2) Equatorial region (St#4-6) is marked by high temperature and lower salinity and chlorophyll in the upper layers. In the eastern side of the transect we found three regions: (3) Cape Verde region (St#7-10), exhibiting lower salinity in the upper layers, with an Oxygen Minimum Zone (OMZ) located between 200 and 700 m depth. The core of the OMZ was observed at stations 8 and 9, between 300 and 400 m depth, with oxygen concentrations of approximately 40 mol·kg^1^. (4) The upwelling region (St#11) is characterized by lower temperature in the upper layer and high levels of chlorophyll influenced by the mesoscale oceanographic structures and the near Mauritanian upwelling. Finally, in the northernmost side of the transect, (5) the Canary Islands region (St#12) show distinct high temperature and relatively high values of salinity and oxygen in the first 200 m of the water column.

### Functional traits database

Complete trait information was available for 191 species, accounting for over 84.5% of the total species identified (for more details on taxonomic identifications, see Fernandez de Puelles et al.^43^,) (Supplementary Table 1). Six functional traits, related to behavior, life history, and morphology, were selected for each species, based on previous compilations^5,10,11,51^ and complemented with data from public databases such as Marine Planktonic Copepods (https://copepodes.obs-banyuls.fr/en). *Body size* (numeric variable, in mm), which reflects energy requirements, was measured from adult females collected during the survey. *Feeding mode* (factor) describes the species’ feeding strategy (ambush, filter, cruise, or mixed). *Myelination* (factor) indicates an ecological adaptation for faster attack or avoidance responses (myelinated or non-myelinated). *Spawning strategy* (factor) relates to the species’ egg-release method (broadcaster or sac-spawning). *Trophic regime* (factor) identifies the species’ role in food webs (carnivore, omnivore, omnivore-carnivore, omnivore-herbivore, and omnivore-detritivore). Finally, *vertical distribution* (ordered factor) indicates the species’ position in the water column (epipelagic, mesopelagic, or bathypelagic) (see Supplementary Table 2 for more details). The species list and their associated functional traits are provided in Supplementary Table 1.

### Measures of taxonomic and functional diversity

Copepods were classified into five taxonomic levels: species, genus, family, superfamily, and order. The taxonomic analysis used the same copepod dataset as the functional analysis to facilitate comparison and included three indices: species richness (*SR*), average taxonomic distinctiveness (*Δ+, AvTD*) and variation in taxonomic distinctiveness (*Λ+, VarTD*)^60^. They were estimated using the *taxondive* function in *vegan* package^61^ and provide insights into taxonomic divergence, facilitating the exploration of diversity patterns and providing information about the processes shaping the regional species assemblage^62^. Higher values of *Δ+* indicate more diverse assemblages and a greater separation. Moreover, high values of *Λ+* suggest that most of species in the assemblage are concentrated in a few taxa, while lower values indicate a more even distribution of species across hierarchical levels^60^.

Functional *α*-diversity was quantified using different indices, with the *alpha.fd.multidim* function in the *mFD* package^63^. In this analysis, Functional Dispersion (*FDis*) represents the average distance of species to the centroid of all species in the multidimensional trait space, i.e., provides information on how species are distributed in the functional space and is linked to niche differentiation and competition level^64,65^. Functional Evenness (*FEve*) measures the uniformity of traits distribution and regularity in the functional space^66^. This index is independent of *SR*, and its values range between 0 and 1. Functional Divergence (*FDiv*) determines the distribution of functional traits in the community and quantifies the functional variability between the different species present^66,67^. This index is also constrained between 0 and 1, being close to 1 when most species exhibit extreme functional traits, and close to 0 when most species have functional traits closer to the centroid of functional space^68^. Functional Specialization (*FSpe*) quantifies the average distinctiveness of all species and is measured as the mean Euclidean distance between each species and the mean position of all the species in the assemblage. Higher values (reaching to 1) indicate that the species are located far from the centroid, exhibiting extreme functional traits^47,69^. Functional Originality (*FOri*) represents the uniqueness of the traits of the threatened species^47,64^.

To examine *β*-diversity patterns across depths and stations, we estimated the contributions of turnover and nestedness to dissimilarity using *Sørensen*’s dissimilarity index^19^ for each haul. Taxonomic *β*-diversity was represented by *TDsor* (overall dissimilarity), *TDsim* (turnover), and *TDsne* (nestedness), while functional *β*-diversity was represented by *FDsor*, *FDsim*, and *FDsne*. These values were calculated using the *beta.pair* and *functional.beta.pair* functions from the *betapart* package^70^. Finally, functional *β*-diversity comparisons across depths and stations were visualized in two-dimensional space using the *beta.fd.multidim* function in the *mFD* package.

### Statistical analysis

A principal coordinates analysis (PCoA) was performed on the functional matrix based on Gower distance^71,72^. The coordinates of the first four axes of the PCoA which minimized the absolute deviation (MAD = 0.06) (Supplementary Figs. 1, 2) were retained to build this space^68,71^ and to determine the species distribution within the functional space. Functional groups (FGs) were identified from this cluster using the *average* method, which generates the lowest distance matrix and provides a better approximation to dissimilarities and representation^73^.

All facets of α- and *β*-diversity were estimated for each station, considering both depth stratum and day/night period. Subsequently, mean values for these factors were calculated according to the study type. *Spearman* correlation was used to evaluate the association between *α-*taxonomic and functional indices. A two-factor nested ANOVA analysis was conducted to examine the spatial and day/night changes in the *α*-diversity facets, with depth as factor (seven levels) and time as nested variable (two levels) within each ecoregion. The upwelling (St#11) and Canary Islands (St#12) ecoregions were excluded due to having only one station. To meet the assumptions of ANOVA, we checked the residuals for normality (Shapiro test, *p* < 0.05) and homogeneity of the variances (Levene’s test, *p* < 0.05). In consequence, a Box-Cox transformation was applied for all facets of taxonomic and functional *α*-diversity, except for *FDiv* and *FOri* indices^74^.

Generalized Additive Models (GAMs)^75^ were used to relate diversity metrics to environmental variables (i.e., chlorophyll, depth, density, fluorescence, salinity, oxygen, temperature, longitude, latitude, day/nighttime). These models were constructed using the *mgcv* package (Wood, 2023). Previously, environmental variables (except for latitude and longitude) were reduced to a set of explanatory variables, ensuring they were not highly correlated. This was achieved using Spearman’s correlation and *VIF* index from *usdm* package^76^ and selecting only those variables with a *VIF* < 3^77,78^. The numerical variables were standardized by subtracting their respective means and dividing by their standard deviations. GAMs were fitted using a Gaussian identity link function with a thin plate regression spline smoother. Generalized cross-validation (GCV) was employed to automatically select the degrees of freedom, constrained by the variable and model specification. The smoothing parameter was estimated using the restricted maximum likelihood method (REML). Model selection was based on the examination of Q-Q plots and residual scatterplots, ensuring that no issues with residual normality or dispersion were detected (Potts & Rose, 2018). Additionally, spatial autocorrelation of the residuals in each final model was assessed using Moran’s I test^79^, with results indicating none to very low spatial autocorrelation. Model evaluation was based on the Akaike’s Information Criterion (AIC). Thus, the ΔAIC value was used (ΔAIC = 0) to find the ‘best’ model, which is the difference between the AIC value for each model and the lowest observed ΔAIC value. Moreover, models with AIC values differing by less than 2 were considered equally plausible^80,32^.

The correlation between taxonomic and functional *β*-diversity, along with their turnover and nestedness components, was assessed separately using a Mantel test with 999 permutations^81^. Comparison of *β*-diversity indices by depth and station were examined with Kruskal–Wallis tests^82^, followed by Dunn’s multiple comparison test with Benjamini-Hochberg correction for post-hoc analysis. Data did not meet the assumptions of normality (Shapiro test, *p* < 0.05) and homogeneity of variance (Barlett’s test, *p* < 0.05). All statistical processes were performed in R environment (R^83^).

## Results

### Functional space and groups

The first four PCoA components selected explained 73% of total variance. The first dimension explained the 26.7% of the variation (Supplementary Fig. 3) and separated species based on the myelination traits (Supplementary Fig. 2). The positive values identified amyelinated species, whereas the negative values indicated myelinated species. The second dimension explained the 21% of the variation (Supplementary Fig. 3), mainly segregating species according to their spawning mode (Supplementary Fig. 2). Sac-spawners species where predominantly located in the positive values, whereas broadcaster species were distributed in the negative values.

**Fig. 2:**
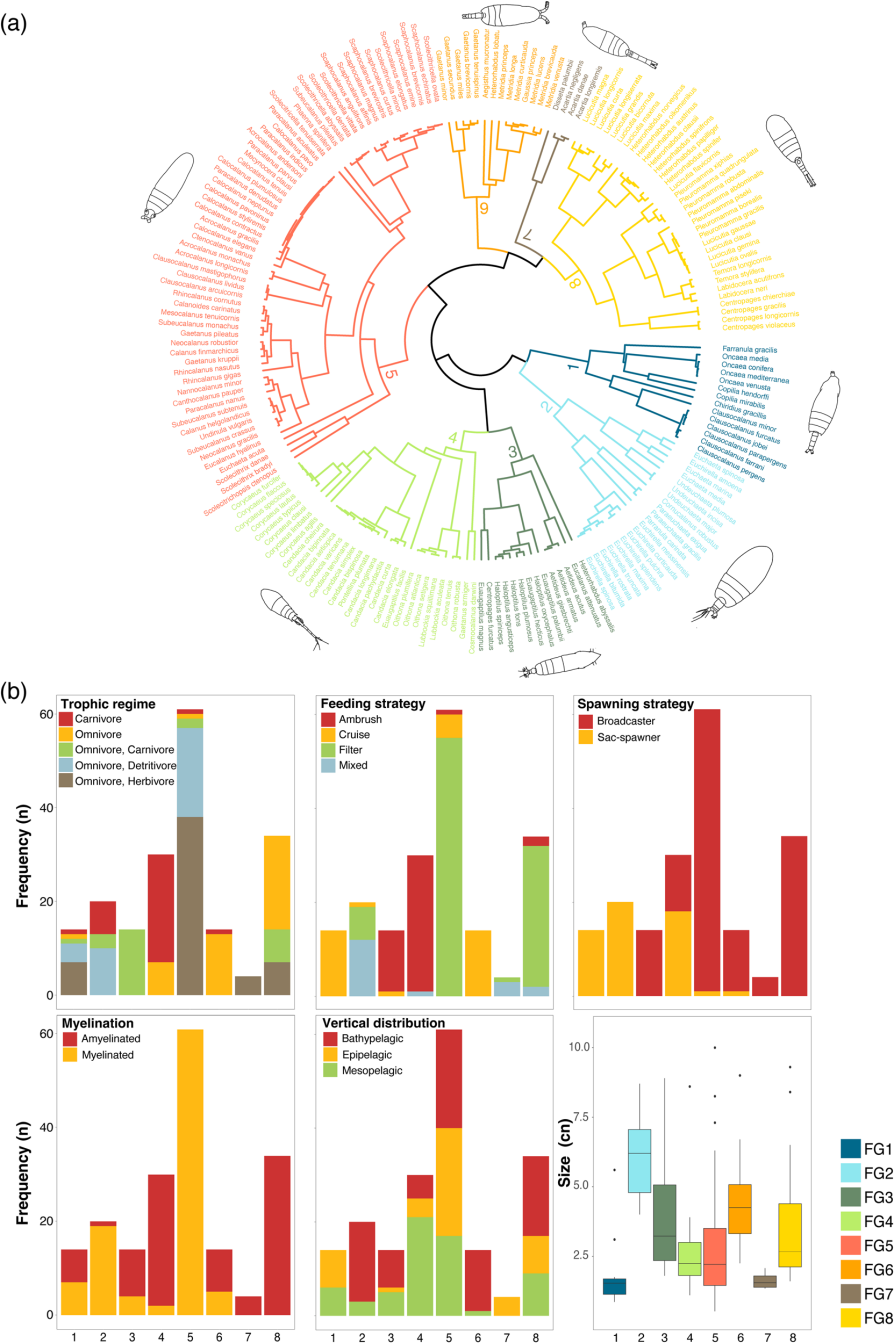
Combination of traits can form distinct copepod functional groups. Functional dendrogramand groups (FG) differentiated across the tropical and equatorial Atlantic Ocean (**a**). Bar plot of the frequency for functional traits by FGs (**b**). Copepod illustrations were taken from Ferrari and Bradley (1993) and Mazzocchi et al. (1995).

**Fig. 3:**
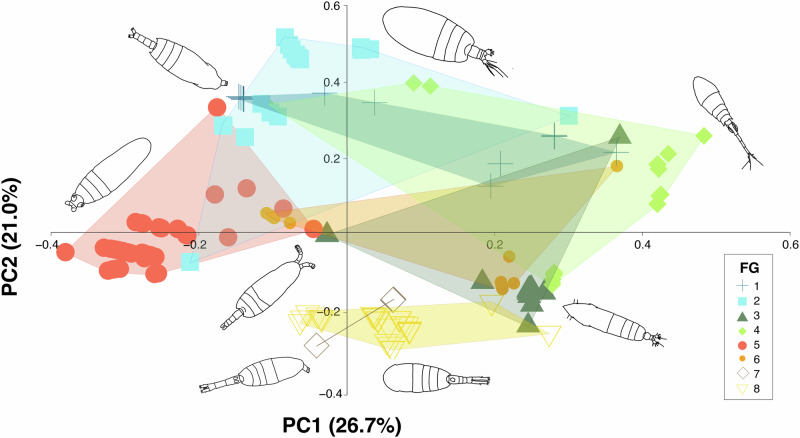
Illustration of the functional space between PC1 and PC2 with the convex hull of each functional group. Copepod illustrations were taken from Ferrari & Bradley (1993) and Mazzocchi et al. (1995).

A total of eight FGs were identified from the cluster analysis (Fig. 2). Species from different FGs were clustered and concentrated primarily in the lower section of the functional space, while at the top, they were more widely distributed (Fig. 3). The number of species composing each FGS and their functional characteristics were as follows:

- FG1 comprised 14 species with size ranging from 0.86 to 5.60 mm, exhibiting traits of omnivore-herbivores and omnivore-detritivores, and utilizing a cruise feeding strategy. All species in this group were sac-spawners displaying both myelinated and amyelinated traits and inhabiting the epipelagic and mesopelagic layers.
- FG2 consisted of 20 species with body size ranging from 4.00 to 8.70 mm. More than half of these species were omnivore–carnivores employing mixed feeding strategies. All inhabit the bathypelagic zone, were sac-spawners, and myelinated.
- FG3 included 14 species ranging from 1.8 to 8.90 mm, characterized as carnivorous species with an ambush feeding strategy. These species predominantly used broadcaster spawning and were amyelinated, inhabiting both the mesopelagic and bathypelagic zones.
- FG4 consisted of 30 species ranging from 1.10 to 8.60 mm with carnivorous trophic regime and ambush feeding strategy. They presented both broadcaster and sac-spawner strategies, were amyelinated, and were prevalent in the mesopelagic layer.
- FG5 represented the largest group with 61 species, varying in body size from 0.52 to 10.00 mm. They exhibited omnivore-herbivore and omnivore-detritivore trophic regimes, employing a filter feeding strategy. All species were broadcaster and myelinated, as well as distributed across the entire water column.
- FG6 comprised 14 species with body sizes ranging from 2.25 to 9.00 mm, characterized as omnivorous species with mixed feeding strategies. They were broadcaster and amyelinated, occurring in the bathypelagic layer.
- FG7 was the smallest group, consisting of four species with body sizes between 1.34 and 2.07 mm. They were omnivore-herbivores employing a mixed feeding strategy, broadcasters, amyelinated, and resided in the epipelagic layer. FG7 was the smallest group, consisting of four species with body sizes between 1.34 and 2.07 mm. They were epipelagic, omnivore herbivores employing a mixed feeding strategy, broadcaster, and amyelinated.
- FG8 included 34 species ranging from 1.60 to 9.30 mm. All were omnivorous using a filter feeding strategy. They were broadcasters with amyelinated characteristics, and predominantly occupying the bathypelagic layer.

### Taxonomic and functional *α*-diversity

**Fig. 4:**
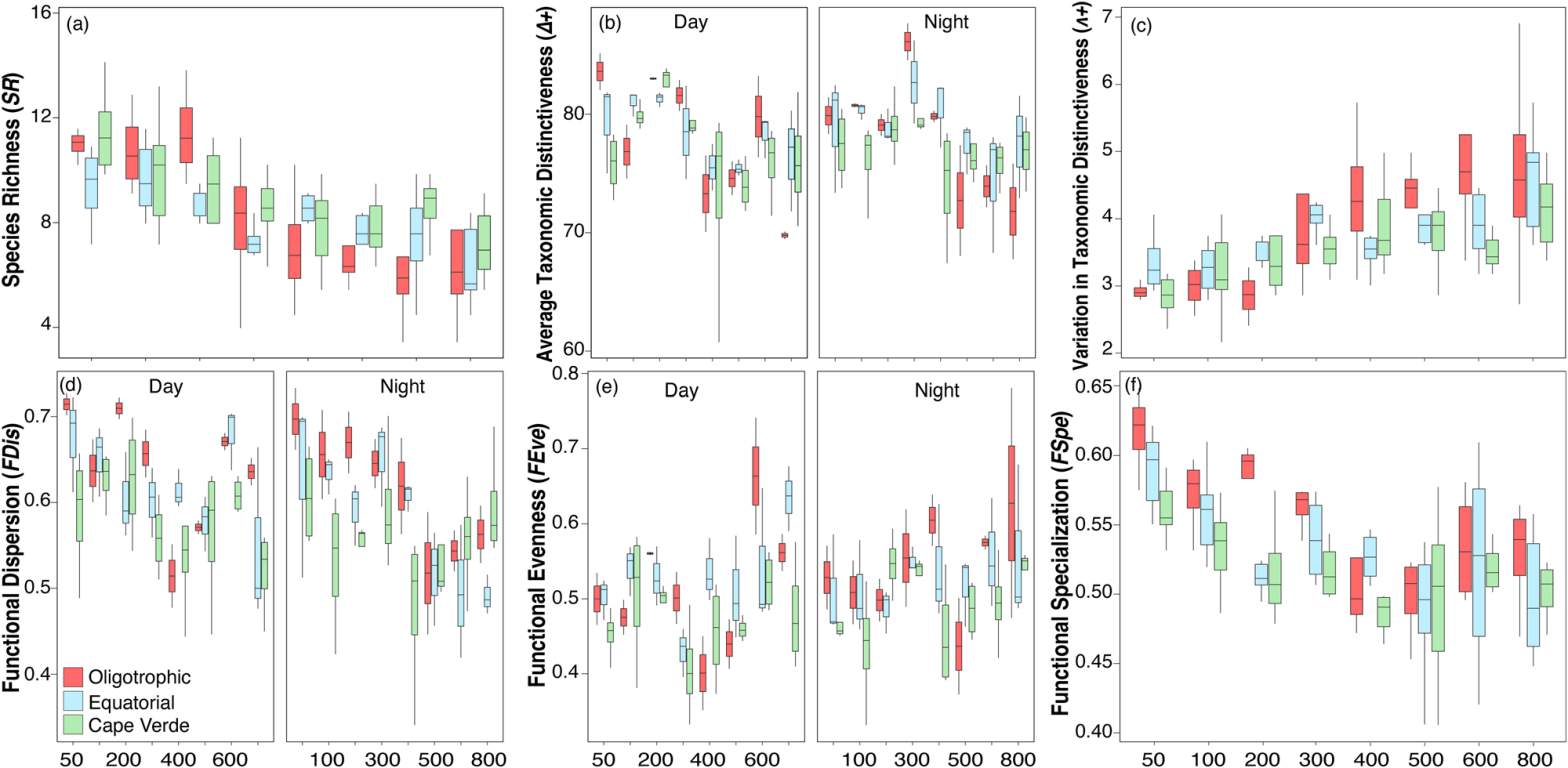
Boxplot of nested ANOVA for significant taxonomic and functional α-diversity indices. In each box plot, the median marks the mid-point of the data and is shown by the line that divides the box into two parts, and the upper and lower whiskers represent scores outside the middle 50% (i.e., the lower 25% of scores and the upper 25% of scores). Colors stand for ecoregions. SR (**a**), *Δ*+ (**b**), *Λ*+ (**c**), *FDis* (**d**), *FEve* (**e**), and *FSpe* (**f**).

The nested ANOVA revealed that all taxonomic facets varied across the depths and ecoregions, with only *Δ+* displaying a significant interaction between time (day/night) and depth (Table 1). Both *SR* and *Δ*+ exhibited similar patterns, with higher values in the upper layers (< 200–300 m) and a steep decline at greater depths, whereas *Λ+* increased with depth. Moreover, *SR* the was lesser in the oligotrophic waters than in the Cape Verde ecoregion, but *Λ+* was higher (Fig. 4a–c). Spatial differences were also found between ecoregions and depth for three functional indices, *FDis*, *FEve* and *FSpe* (Table 1). *FDis* showed a day/night pattern similar to *Δ*+, *FEve* acquired higher values comparable in deeper waters and *FSpe* followed a pattern comparable with *SR*. Moreover, *FDis* and *FSpe* reached values higher in oligotrophic waters (Fig. 4d–f). In general, a strong correlation was observed between taxonomic and functional *α-*diversity indices (Supplementary Table 3), except for the *FDiv* and *FOri* indices.

**Table 1:** Nested ANOVA on the effects of region, depth and time in taxonomic and functional α-diversity indices

Taxonomic		*SR*		*Δ+*		*Λ+*	
	df	*F*	*p*	*F*	*p*	*F*	*p*
Region	2	3.761	*	4.211	*	3.778	*
Region: depth	21	5.077	***	5.046	***	5.055	***
Region: time	3	2.470	ns	0.387	ns	2.466	ns
Region: depth and time	21	1.312	ns	1.979	*	1.323	ns
Residuals	96						
**Functional**		***FDis***		***FDiv***		***FEve***	
	df	*F*	*P*	*F*	*P*	*F*	*P*
Region	2	10.370	***	0.491	ns	9.531	***
Region: depth	21	2.947	***	0.927	ns	2.306	**
Region: time	3	3.106	*	2.055	ns	1.310	ns
Region: depth and time	21	1.764	*	0.954	ns	1.857	*
Residuals	96						
**Functional**		***FOri***		***FSpe***			
	df	*F*	*P*	*F*	*P*		
Region	2	1.619	ns	8.948	***		
Region: depth	21	1.443	ns	4.281	***		
Region: time	3	0.805	ns	2.224	ns		
Region: depth and time	21	0.875	ns	1.545	ns		
Residuals	96						

*df* degree of freedom, *FDis* functional disparity, *FDiv* functional divergence, *FEve* functional evenness, *FOri* functional originality, *FSpe* functional specialization, *SR* species richness, *ns* no significative; *= *p* < 0.05, ** = *p* < 0.01, ***= *p* < 0.001, *Δ*+, average taxonomic distinctiveness; *Λ*+, variation in taxonomic distinctiveness.

**Table 2:** Estimated coefficients for selected environmental and spatial variables from generalized additive model (GAMs) for overall taxonomic and functional α-diversity indices

GAM models	Variables	*p*	*AIC*	*Var* (%)	*R*^2^_adj_
*SR* ~ s(Lat,Lon) + log(Depth) + Time +~ s(Chl) + s(O_2_)			1061.55	39.5	0.344
	s(Chl)	ns			
	s(O_2_)	ns			
	**s(Lat,Lon)**	**<** **0.001**			
	**log (Depth)**	**<** **0.001**			
	Time	ns			
*Δ*+ ~ s(Lat, Lon) + log(Depth) + Time +~ s(Chl) + s(O_2_)			9251.26	38.9	0.276
	**s(Chl)**	**0.002**			
	**s(O**_**2**_**)**	**0.002**			
	**s(Lat,Lon)**	**<** **0.001**			
	**log (Depth)**	**<** **0.001**			
	Time	ns			
*Λ+* ~ s(Lat, Lon) + log(Depth) + Time +~ s(Chl) + s(O_2_)			343.18	48.5	0.422
	s(Chl)	ns			
	s(O_2_)	ns			
	**s(Lat,Lon)**	**<** **0.001**			
	**log (Depth)**	**<** **0.001**			
	Time	ns			
*FDis* ~ s(Lat,Lon) + log(Depth) + Time +~ s(Chl) + s(O_2_)			−456.38	28.2	0.240
	s(Chl)	ns			
	s(O_2_)	ns			
	s(Lat,Lon)	ns			
	**log (Depth)**	**0.014**			
	**Time**	**0.012**			
*FDiv* ~ s(Lat,Lon) + log(Depth) + Time +~ s(Chl) + s(O_2_)			-	-	-
	s(Chl)	ns			
	s(O_2_)	ns			
	s(Lat,Lon)	ns			
	log (Depth)	ns			
	Time	ns			
*FEve* ~ s(Lat,Lon) + log(Depth) + Time +~ s(Chl) + s(O_2_)			−453.31	21.8	0.190
	s(Chl)	ns			
	**s(O**_**2**_**)**	**0.014**			
	**s(Lat,Lon)**	**<** **0.001**			
	**log (Depth)**	**<** **0.001**			
	**Time**	**0.047**			
*FOri* ~ s(Lat,Lon) + log(Depth) + Time +~ s(Chl) + s(O_2_)			−710.28	15.8	0.115
	s(Chl)	ns			
	**s(O**_**2**_**)**	**<** **0.001**			
	s(Lat,Lon)	Ns			
	**log (Depth)**	**<** **0.001**			
	Time	ns			
*FSpe* ~ s(Lat,Lon) + log(Depth) + Time +~ s(Chl) + s(O_2_)			−649.27	40.4	0.364
	s(Chl)	ns			
	**s(O**_**2**_**)**	**0.012**			
	s(Lat,Lon)	ns			
	**log (Depth)**	**0.002**			
	**Time**	**0.017**			

*p* probability level of significance (ns = non significative), *AIC* Akaike’s information criterion, *Var (%)* variance explained; *R*^*2*^_*adj*_ determination coefficient adjusted.

Significant variables are highlighted in bold.

**Fig. 5:**
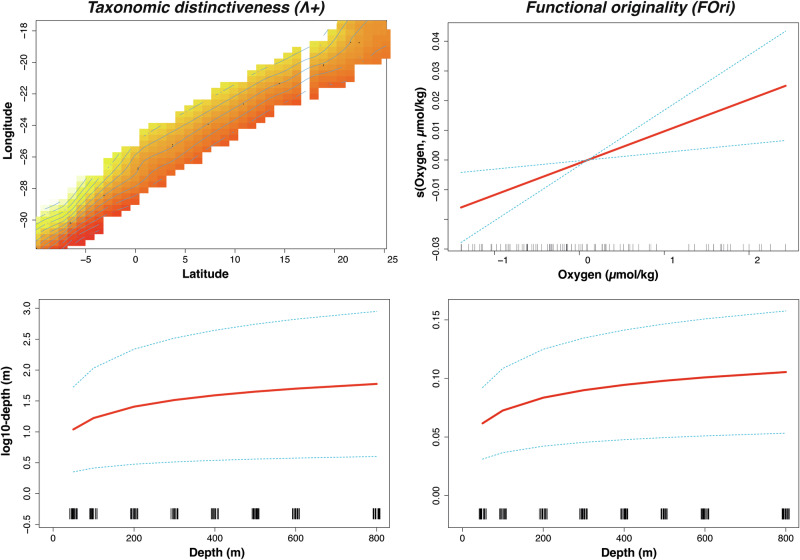
Generalized additive model (GAM) response curves for variation in taxonomic distinctiveness (left) and functional originality (right). Dashed lines indicate that 95% confidence intervals for each response curve. Rug plots are displayed at the bottom of each subplot.

Due to the high correlation between some environmental variables (Supplementary Table 4) and to avoid collinearity, only the oxygen, and chlorophyll were retained based on the *VIF* analysis. All GAM models, except for FDiv, demonstrated significant relationships with environmental variables (Table 2). Depth was the only consistently significant variable across all models, while latitude and longitude were particularly important in the taxonomic models. Additionally, the time factor (day/night) influenced the functional patterns. The index Λ+ yielded the best-fit taxonomic GAM (AIC = 343.18), which incorporated the interaction between longitude, latitude, and depth (Fig. 5), explaining 48.5% of the variance. In contrast, the FOri index provided the best-fit functional diversity GAM (AIC = −710.28), with depth and oxygen as significant factors, though it explained only 15.8% of the variance (Fig. 5). Notably, the FSpe model explained 40.4% of the variance and included the time factor (Table 2).

### Taxonomic and functional *β*-diversity

**Fig. 6:**
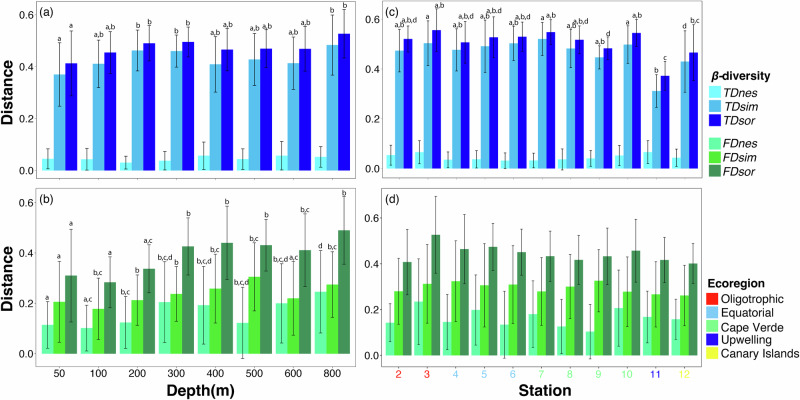
Comparison of β-diversity across the depth and stations. Taxonomic diversity (**a, c**) and functional diversity (**b, d**). Bars with different lowercase letters indicate significant differences (*P* < 0.05) among groups. *FDsor*, *FDsim*, and *FDsne* are overall, turnover and nestedness of functional *β*-diversity; *TDsor*, *TDsim*, and *TDsne* represent the overall, turnover and nestedness of taxonomic *β*-diversity. Error bars refer to standard deviation of the mean.

Functional dissimilarity, along with its turnover and nestedness components, was significantly correlated with the corresponding taxonomic dissimilarity (*p* < 0.05), exhibiting a stronger relationship in the turnover component (Mantel’s r = 0.514) compared to the nestedness component (Mantel’s r = 0.417). Overall taxonomic and functional *β*-diversity (*TDsor*, *FDsor*), as well as their turnover and nestedness components (*TDsim*, *FDsim*, *FDnes*), were significantly lower in shallower strata ( < 300 m), particularly for functional diversity (Fig. 6a, b). Notably, only the taxonomic nestedness component (*TDnes*) did not show significant changes.

**Fig. 7:**
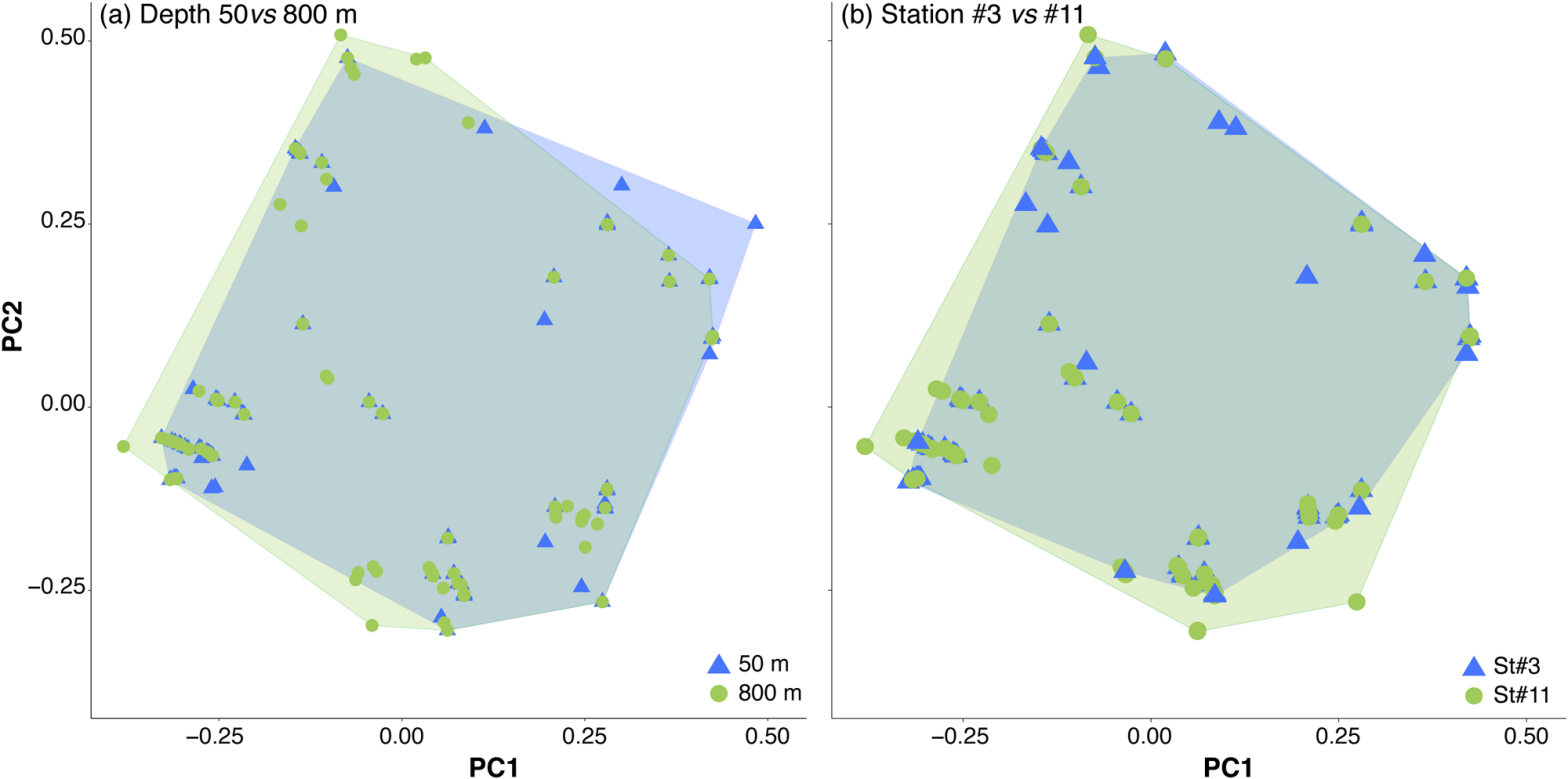
Illustrative comparison of the multidimensional functional space for functional β-diversity. Depth comparison between 50 and 800 m (**a**) and between oligotrophic (St#3) and upwelling regions (St#11) (**b**).

Taxonomic and functional *β*-diversity displayed distinct patterns; taxonomic *β*-diversity was predominantly influenced by the turnover component, which accounted for more than 87.2% of total dissimilarity, while functional *β*-diversity was more evenly distributed (*FDsim* ranged from 51.5% to 71.7%). However, species replacement did not coincide with substantial functional changes, resulting in an overlap of functional space (Fig. 7a).

When analyzed by station, the upwelling station (St#11) exhibited the lowest values for overall taxonomic *β*-diversity (*TDsor*) and the turnover component (*TDsim*), while the nestedness component (*TDnes*) remained constant (Fig. 6c). Although the turnover component was the primary contributor to overall diversity (*TDsim* and *FDsim* exceeding 83.4% and ranging from 57.1% to 76.7%, respectively), the nestedness component had a more pronounced influence. The three facets of functional *β*-diversity did not vary significantly between stations, though a slight decrease was observed from oligotrophic (St#2-3) to upwelling (St#11) ecoregions (Fig. 6d). Consequently, while copepod assemblage composition differed in waters influenced by African upwelling, the functional space also showed considerable overlap (Fig. 7b).

## Discussion

This study provides evidence for the shifts in both taxonomic and functional *α*- and *β-*diversity within the copepod communities across the ecoregions of the Central Atlantic Ocean. Our results reveal a high correlation between alpha taxonomic and functional metrics, indicating that modelling functional diversity hinges upon the species composition of copepod communities. Spatial gradients, including latitude, longitude, and depth, emerge as the primary drivers influencing these diversity shifts. The analysis of *β-*diversity revealed a correlation between taxonomic and functional diversity, with a considerable variation in species composition across ecoregions and depth exhibiting similar functions. In contrast, the nestedness component was more noticeable in the functional diversity.

The functional groups described in the present study differed from those identified by Benedetti et al.^5^. This discrepancy could be attributed to differences in spatial scale (regional *versus* global), species composition, and the functional analysis method applied. While Benedetti et al.^5^ utilized a factor analysis of mixed data (FAMD), our approach relied on methodologies based on multidimensional space used a factor analysis of mixed data (FAMD), our approach relied on multidimensional space methodology^68^. For instance, in the study of Benedetti et al.^5^*Clausocalanus* was placed in FG1 and *Oncaea* in FG2, reflecting their trophic regime disparity (omnivore-herbivore and detritivore, respectively). However, our findings grouped them together within the same functional group (FG1), prioritizing their classification as generalist species and their cruising feeding strategy, despite their low species count. Additionally, we observed a higher number of species within the FG5, which were dispersed in Benedetti et al.^5^ separating *Calocalanus* and *Paracalanus* from *Scaphocalanus*. Furthermore, we grouped *Pleuromamma* with *Lucicutia*, and *Heterorrhabdus* in the FG8, whereas Benedetti et al.^5^ associated this genus with *Gaetanus* and *Metridia*. Therefore, conducting a direct comparison between the two studies might not be the most adequate approach, and instead, the most relevant issue is the ecological patterns derived from each study.

The abundance and distribution of copepods across the Atlantic ecoregions have been described in several studies^5,43,84^. They concluded that *Oncaea* and *Oithona* are the most common genera, with especially *Oncaea venusta* and *Oithona plumifera*, followed by *Clausocalanus* with *C. furcatus*. Oncaeid species dominates the mesopelagic and bathypelagic layers across all ecoregions, with the highest abundance observed in the upwelling area. In contrast, *Clausocalanus* spp. inhabit the epipelagic layer, and although common, reach greater abundance in the southern areas (ref. ^85^ Bendetti et al. 2022, Fernandez de Puelles et al.^43^). The FG5 was the most represented group, encompassing 61 species from various genera such as *Scaphocalanus*, *Calocalanus*, *Scolecithricella*, and *Paracalanus* spp. While most genera maintained a consistent and low abundance across ecoregions ( < 2%), the genus *Paracalanus*, represented by *P. parvus* and *P. indicus*, exhibited a high abundance in the upper layers of cold and nutrient-rich waters, particularly in Cape Verde and upwelling ecoregions^43^. This could be linked to filter-feeding mode and the broadcaster spawning strategy, which requires lower energy demand. Certain functional groups may be more closely associated with specific oceanographic conditions. For example, FG8, represented by *Pleuromamma* spp. and *Temora* spp., was more abundant in the upwelling ecoregion (Fernandez de Puelles et al. 2023). However, this group comprised omnivore-detritivore and omnivore-herbivore species, allowing them to have a wider distribution and be better adapted to different environments. FG4, characterized by *Oithona* spp. and *Corycaeus* spp., showed slightly greater abundance in the oligotrophic and equatorial ecoregions ^43^. This distribution is influenced by competition under resource-limited conditions, leading to a higher presence of carnivore species utilizing ambush-feeding modes, which requires less energy (Kiørboe et al. 2011). Moreover, these conditions can promote carnivorous strategies, including cannibalism^50^. Therefore, shifts in community composition seems to be associated with a greater frequency of certain functional traits^5,10,44,86^.

We did not find that body size was significant for any of the principal components. However, previous studies have shown that this functional trait is related to temperature, with smaller copepods associated with warmer waters and larger copepods in cold waters^10,44^. In contrast, we identified myelination as key trait, which may be linked to habitat preference. This could be explained by the fact that myelination is frequently associated with feeding mode and size^5^. Myelinated copepods have a lipid-rich myelin sheath around their nerves, enabling faster reaction times and thus more efficient feeding or escape behaviours^87^. The myelination exhibits the same spatial patterns as body size and feeding mode. Amyelinated and small species mainly occurred in the tropical gyres, whereas large, myelinated copepods predominated in polar regions^5^.

Diversity appeared to be closely linked to a strong stratified water column^88,89^ and to the composition of microzooplankton^90^. Our findings revealed greater values of species richness, taxonomic distinctiveness, functional dispersion and specialization in the shallower layers. Furthermore, changes in diel vertical movements (DVM) were detected taxonomically (average distinctiveness) and functionally (dispersion and evenness), revealing that numerous species occupy a wider ecological niche. Fernandez de Puelles et al.^43^, pointed out the presence of non-migrant species within the epipelagic layer (e.g., *Paracalanus*, *Clausocalanus*, *Calocalanus*, and *A. danae*), alongside deepwater species that migrate to this layer during the night (e.g., *Pleuromamma*, *Euchirella*, Subeucalanus, *Rhincalanus*). The non-migrant species were grouped together (FG5) except for *Acartia* spp. (FG7). In the case of migrant species, they were assigned to different FGs. *Euchirella* was the most dominant genera in FG2, *Rhincalanus* and *Subeucalanus* were in FG5, and *Pleurommama* was important in FG8.

It is a well-established fact that the zooplankton distribution is influenced by factors such as food availability^2,91^, environmental conditions (oxygen concentration)^92^, water column stratification (Longhutst, 1985^93^), and the distribution of the water masses^94^. The mixed GAMs models pointed out spatial factors (latitude, longitude, and depth) as the key for understanding both the taxonomic and functional composition for most indices. Interestingly, functional originality and specialization models did not show a clear geographical pattern, although the level of differentiation and competition level between species (functional dispersion) was higher in oligotrophic waters, occupying extreme zones within multidimensional space (functional specialization). In contrast, these indices exhibit a sensitivity to oxygen fluctuations, particularly within the OMZ. Previous studies observed aggregations of copepods (e.g., *Eucalanus*, *Subeucalanus*, *Paraeucalanus*, and *Pleuromamma*) in OMZs^95–97^ suggesting a metabolic slowdown under such conditions. This indicates that the Cape Verde ecoregion harbours more specialized copepods. However, Conversely, chlorophyll did not emerge as a significant variable in any of the functional indices but showed significance with the average taxonomic. Regions with higher productivity may have greater species richness, but a low impact in the functional *α-*diversity at the local scale^7,10^. This occurrence might be attributed to the thorough examination of correlations among environmental variables and the integration of spatial variables, a facet often overlooked in alternative studies.

We observed a correlation between taxonomic and functional *β*-diversity, though with some differences. Taxonomic *β*-diversity was primarily driven by the turnover component, remaining stable across depths and stations. In contrast, the nestedness component became more prominent in functional *β*-diversity, particularly at depths greater than 300 m. This may be attributed to oxygen acting as an environmental filter (niche filtering hypothesis^48^), with the oxygen minimum zone (OMZ) occurring between 200 and 700 m depth^58^. Species contributing to the nestedness component tend to occupy the periphery of functional spaces, displaying the most extreme trait combinations, which influences the overlap between convex hulls. These species are predominantly bathypelagic, belonging to FG2 (positive PC2 values, Fig. 7a), FG8 (negative PC2 values, Fig. 7a), and FG5 (negative PC1 values, Fig. 7a). This observation may partially support the idea of a buffering mechanism in ecosystems, where species replacements occur with minimal functional differentiation. This allows new species to maintain similar ecological roles despite taxonomic changes, while staying within the convex hull boundaries.

Studies on zooplankton, including copepods, have shown that environmental factors drive differences in functional traits (refs. ^8,44^ Tang et al. 2021^5^). Our results, however, did not reveal significant differences in overall diversity or its components across stations, suggesting that these communities maintain a well-defined structure, regardless of taxonomic differentiation. The wide distribution of species and functional groups that complement ecological niche space (Fig. 7b) likely explains why functional *β*-diversity remained stable. In fact, the impact of species loss or gain on ecosystem functioning is influenced by the degree of trait overlap among species within a community^98^.

Theoretically, communities with lower taxonomic diversity (e.g., St#11) might be expected to show increased functional *β*-diversity^17,99,100^, but this was not observed in our study. Interestingly, taxonomic and functional patterns appeared spatially homogeneous and strongly correlated. It is possible that the large biogeographic scale influences local environmental constraints or the scope of species competition^17^. Although functional redundancy (defined as similarity in functional roles among species) was not directly estimated, this homogenization suggests a certain degree of redundancy. This supports the hypothesis that ecological communities with more functionally redundant species tend to exhibit higher resilience and stability in both community structure and ecological function over time^101,102^.

In conclusion, to the best of our knowledge, this study represents the first comprehensive examination of both α- and *β-*diversity of copepods evaluating both functional and taxonomic approaches in the Atlantic Ocean. Our analysis revealed eight functional groups of copepods strongly associated with spatial gradients of environmental conditions. The incorporation of *β-*diversity in this study was essential to unveil a high functional similarity in the studied area, characterized by species turnover without significant functional differentiation within ecoregions. Moreover, our findings underscore the importance of considering both *α*- and *β*-diversity, utilizing taxonomic and functional approaches, as they yield complementary insights into community structure. Therefore, future studies should include abundance data to obtain even more robust and specific results, facilitating a more in-depth analysis of community structure. Taxonomic studies under microscopy are imperative for species identification and community characterization. In addition, accurate species identification through classical and/or molecular methods are imperative to characterize the community structure and functioning.

## Supplementary information

Supplementary Information

## Data Availability

Data is provided within the manuscript or supplementary information files.
