# Supplementary material for: Modelling the alpha and beta diversity of copepods across tropical and subtropical Atlantic ecoregions
Source: NPJ Biodivers. 2025 Jan 31;4:3. doi: 10.1038/s44185-025-00073-x (PMC11785948; doi:10.1038/s44185-025-00073-x)
Supplement: Supplementary file 1 — Supplementary Information [file 44185_2025_73_MOESM1_ESM.docx]

**Supplementary material**

**Modelling the alpha and beta diversity in the community of copepods across tropical and subtropical Atlantic ecoregions: a taxonomic and functional approach**

**
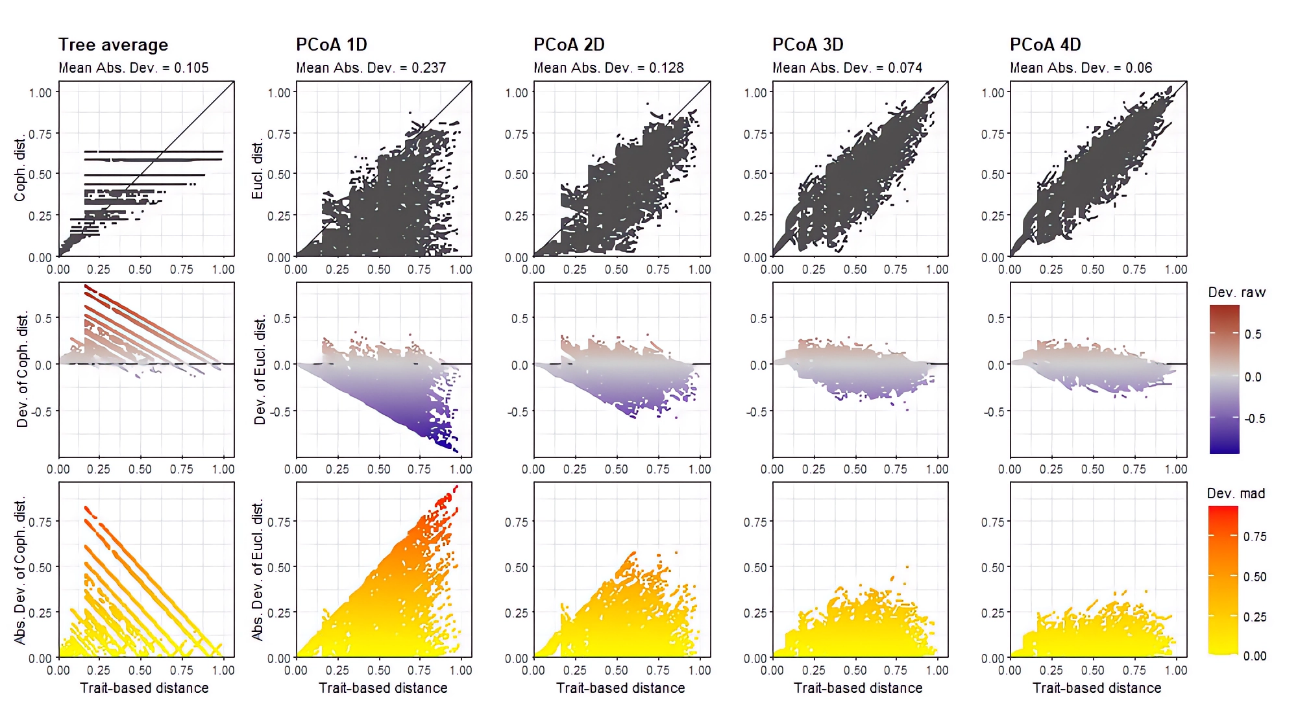
**

**Supplementary Figure 1.** Scatterplots illustrating the quality of the multidimensional trait space with increasing number of PCoA axes. The upper line of plots shows species functional distances in the multidimensional space. In the middle it is shown the deviation of species distances in the functional space compared to trait-based distances. The plots in the down side of the figure show the absolute deviation of the distance in the functional space. The mean absolute deviations (MADs) between the original trait-based distances and the Euclidean distances in the functional space are indicated at the top for each number of PCoA dimensions.

**
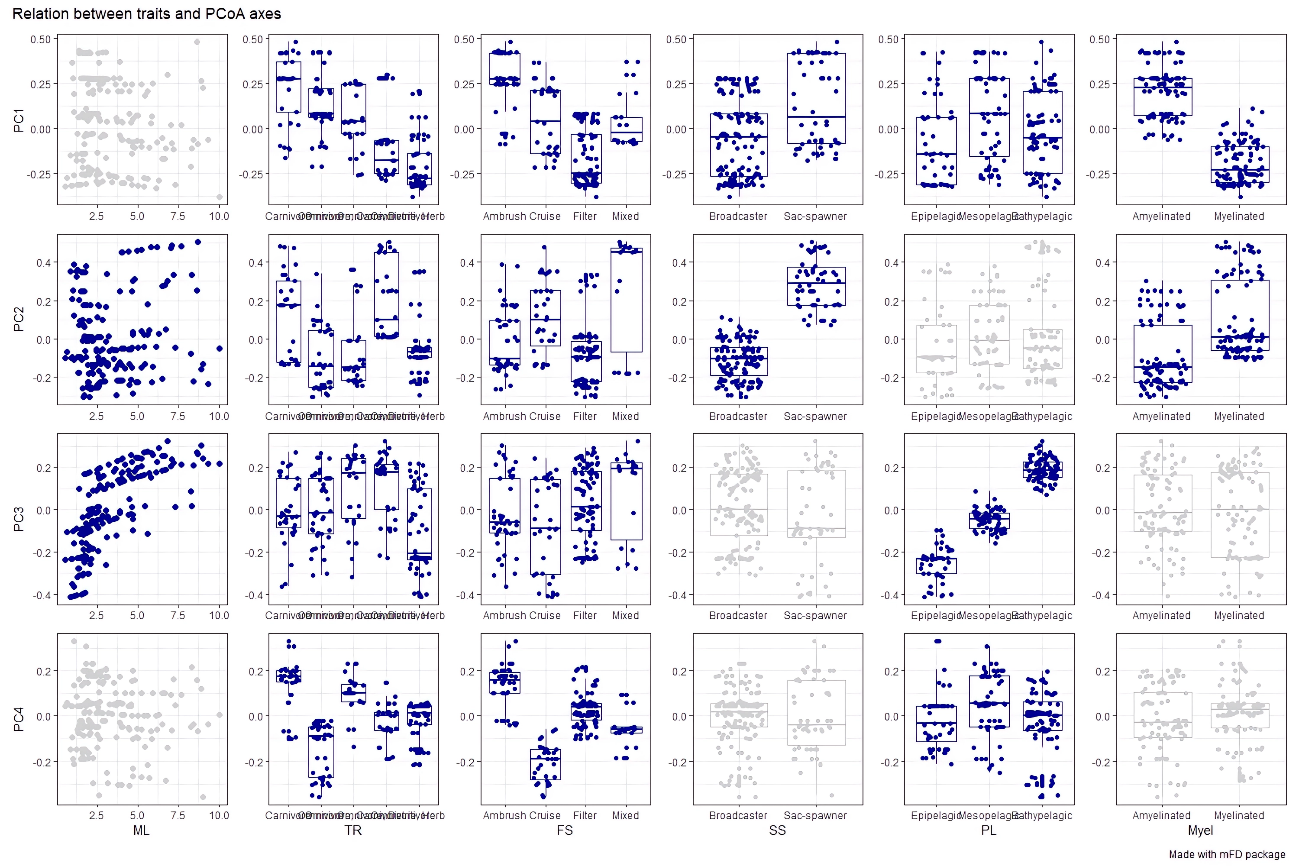
**

**Supplementary Figure 2** Boxplot illustrating the direction of the correlation between individual traits and PCoA axes. Significant trait-PCoA axes correlations are plotted in blue, while non-significant correlations are plotted in grey.


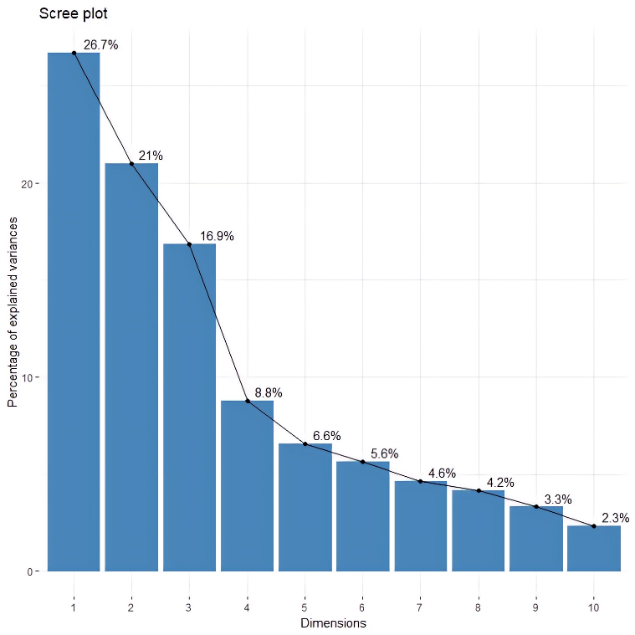


**Supplementary Figure 3** Scree plot illustrating the eigenvalues of the principal components.

**Supplementary Table 1** List of the 226 copepods species and their allocated functional trait. NA, species without complete information.

| **Species** | **Body size**  **(mm)** | **Trophic regime** | **Feeding mode** | **Spawning Strategy** | **Vertical distribution** | **Myelination** |
| --- | --- | --- | --- | --- | --- | --- |
| *Acartia danae* | 1.34 | Omnivore, Herbivore | Mixed | Broadcaster | Epipelagic | Amyelinated |
| *Acartia longiremis* | 1.40 | Omnivore, Herbivore | Mixed | Broadcaster | Epipelagic | Amyelinated |
| *Acartia negligens* | 2.07 | Omnivore, Herbivore | Mixed | Broadcaster | Epipelagic | Amyelinated |
| *Acrocalanus andersoni* | 1.30 | Omnivore, Herbivore | Filter | Broadcaster | Epipelagic | Myelinated |
| *Acrocalanus gracilis* | 1.80 | Omnivore, Herbivore | Filter | Broadcaster | Epipelagic | Myelinated |
| *Acrocalanus longicornis* | 1.55 | Omnivore, Carnivore | Filter | Broadcaster | Epipelagic | Myelinated |
| *Acrocalanus monachus* | 1.10 | Omnivore, Carnivore | Filter | Broadcaster | Epipelagic | Myelinated |
| *Aegisthus mucronatus* | 2.60 | Omnivore | Cruise | Sac-spawner | Bathypelagic | Amyelinated |
| *Aetideopsis carinata* | NA | NA | NA | NA | NA | NA |
| *Aetideopsis pseudoarmatus* | NA | NA | NA | NA | NA | NA |
| *Aetideopsis rostrata* | NA | NA | NA | NA | NA | NA |
| *Aetideus acutus* | 1.80 | Omnivore, Carnivore | Ambrush | Broadcaster | Mesopelagic | Myelinated |
| *Aetideus armatus* | 2.25 | Omnivore, Carnivore | Ambrush | Broadcaster | Mesopelagic | Myelinated |
| *Aetideus giesbrechti* | 2.2 | Omnivore, Carnivore | Ambrush | Broadcaster | Mesopelagic | Myelinated |
| *Amallothrix farrani* | NA | NA | NA | NA | NA | NA |
| *Amallothrix valida* | NA | NA | NA | NA | NA | NA |
| *Archescolecithix auropecten* | NA | NA | NA | NA | NA | NA |
| *Arietellus plumifer* | NA | NA | NA | NA | NA | NA |
| *Arietellus simplex* | NA | NA | NA | NA | NA | NA |
| *Augaptilus glacialis* | NA | NA | NA | NA | NA | NA |
| *Augaptilus longicaudatus* | NA | NA | NA | NA | NA | NA |
| *Augaptilus megalurus* | NA | NA | NA | NA | NA | NA |
| *Calanoides carinatus* | 4.00 | Omnivore, Herbivore | Filter | Broadcaster | Bathypelagic | Myelinated |
| *Calanus finmarchicus* | 5.00 | Omnivore, Herbivore | Filter | Broadcaster | Bathypelagic | Myelinated |
| *Calanus helgolandicus* | 3.5 | Omnivore, Herbivore | Filter | Broadcaster | Mesopelagic | Myelinated |
| *Calocalanus contractus* | 0.91 | Omnivore, Herbivore | Filter | Broadcaster | Epipelagic | Myelinated |
| *Calocalanus elegans* | 0.52 | Omnivore, Herbivore | Filter | Broadcaster | Epipelagic | Myelinated |
| *Calocalanus neptunus* | 0.96 | Omnivore, Herbivore | Filter | Broadcaster | Epipelagic | Myelinated |
| *Calocalanus pavo* | 1.5 | Omnivore, Herbivore | Filter | Broadcaster | Epipelagic | Myelinated |
| *Calocalanus pavoninus* | 0.97 | Omnivore, Herbivore | Filter | Broadcaster | Epipelagic | Myelinated |
| *Calocalanus plumulosus* | 1.34 | Omnivore, Herbivore | Filter | Broadcaster | Epipelagic | Myelinated |
| *Calocalanus styliremis* | 0.95 | Omnivore, Herbivore | Filter | Broadcaster | Epipelagic | Myelinated |
| *Calocalanus tenuis* | 1.31 | Omnivore, Herbivore | Filter | Broadcaster | Epipelagic | Myelinated |
| *Candacia aetiopica* | 3.03 | Carnivore | Ambrush | Broadcaster | Mesopelagic | Amyelinated |
| *Candacia bipinnata* | 3.16 | Carnivore | Ambrush | Broadcaster | Mesopelagic | Amyelinated |
| *Candacia bispinosa* | 2.16 | Carnivore | Ambrush | Broadcaster | Mesopelagic | Amyelinated |
| *Candacia cheirura* | 3.12 | Carnivore | Ambrush | Broadcaster | Mesopelagic | Amyelinated |
| *Candacia curta* | 2.90 | Carnivore | Ambrush | Broadcaster | Bathypelagic | Amyelinated |
| *Candacia elongata* | 3.50 | Carnivore | Ambrush | Broadcaster | Bathypelagic | Amyelinated |
| *Candacia longimana* | 3.9 | Carnivore | Ambrush | Broadcaster | Mesopelagic | Amyelinated |
| *Candacia pachydactila* | 2.60 | Carnivore | Ambrush | Broadcaster | Bathypelagic | Amyelinated |
| *Candacia simplex* | 2.32 | Carnivore | Ambrush | Broadcaster | Mesopelagic | Amyelinated |
| *Candacia tenuimana* | 2.40 | Carnivore | Ambrush | Broadcaster | Mesopelagic | Amyelinated |
| *Candacia varicans* | 2.74 | Carnivore | Ambrush | Broadcaster | Mesopelagic | Amyelinated |
| *Canthocalanus pauper* | 1.75 | Omnivore, Herbivore | Filter | Broadcaster | Mesopelagic | Myelinated |
| *Centraugaptilus rattrayi* | NA | NA | NA | NA | NA | NA |
| *Centropages chierchiae* | 2.1 | Omnivore | Ambrush | Broadcaster | Epipelagic | Amyelinated |
| *Centropages furcatus* | 1.92 | Omnivore, Carnivore | Ambrush | Broadcaster | Epipelagic | Amyelinated |
| *Centropages gracilis* | 2.16 | Omnivore | Ambrush | Broadcaster | Epipelagic | Amyelinated |
| *Centropages longicornis* | 2.00 | Omnivore | Mixed | Broadcaster | Epipelagic | Amyelinated |
| *Centropages violaceus* | 2.24 | Omnivore | Mixed | Broadcaster | Epipelagic | Amyelinated |
| *Chiridius gracillis* | 3.10 | Omnivore | Cruise | Sac-spawner | Mesopelagic | Myelinated |
| *Chirundina streetsii* | NA | NA | NA | NA | NA | NA |
| *Clausocalanus arcuicornis* | 1.62 | Omnivore, Herbivore | Cruise | Broadcaster | Epipelagic | Myelinated |
| *Clausocalanus farrani* | 1.22 | Omnivore, Herbivore | Cruise | Sac-spawner | Epipelagic | Myelinated |
| *Clausocalanus furcatus* | 1.75 | Omnivore, Herbivore | Cruise | Sac-spawner | Epipelagic | Myelinated |
| *Clausocalanus jobei* | 1.56 | Omnivore, Herbivore | Cruise | Sac-spawner | Epipelagic | Myelinated |
| *Clausocalanus lividus* | 1.98 | Omnivore, Herbivore | Cruise | Broadcaster | Epipelagic | Myelinated |
| *Clausocalanus mastigophorus* | 1.94 | Omnivore, Herbivore | Cruise | Broadcaster | Epipelagic | Myelinated |
| *Clausocalanus minor* | 1.30 | Omnivore, Carnivore | Cruise | Sac-spawner | Epipelagic | Myelinated |
| *Clausocalanus parapergens* | 1.65 | Omnivore, Herbivore | Cruise | Sac-spawner | Epipelagic | Myelinated |
| *Clausocalanus pergens* | 0.86 | Omnivore, Herbivore | Cruise | Sac-spawner | Epipelagic | Myelinated |
| *Copilia hendorffi* | 1.10 | Omnivore, Herbivore | Cruise | Sac-spawner | Epipelagic | Amyelinated |
| *Copilia lata* | NA | NA | NA | NA | NA | NA |
| *Copilia mirabilis* | 5.60 | Omnivore, Herbivore | Cruise | Sac-spawner | Mesopelagic | Amyelinated |
| *Cornucalanus robustus* | 5.80 | Carnivore | Cruise | Sac-spawner | Bathypelagic | Myelinated |
| *Corycaeus agilis* | 8.60 | Carnivore | Ambrush | Sac-spawner | Bathypelagic | Amyelinated |
| *Corycaeus clausi* | 1.40 | Carnivore | Ambrush | Sac-spawner | Bathypelagic | Amyelinated |
| *Corycaeus flaccus* | 1.82 | Carnivore | Ambrush | Sac-spawner | Mesopelagic | Amyelinated |
| *Corycaeus furcifer* | 1.89 | Carnivore | Ambrush | Sac-spawner | Mesopelagic | Amyelinated |
| *Corycaeus lautus* | 2.1 | Carnivore | Ambrush | Sac-spawner | Mesopelagic | Amyelinated |
| *Corycaeus limbatus* | 3.05 | Carnivore | Ambrush | Sac-spawner | Epipelagic | Amyelinated |
| *Corycaeus speciosus* | 1.64 | Carnivore | Ambrush | Sac-spawner | Mesopelagic | Amyelinated |
| *Corycaeus typicus* | 2.55 | Carnivore | Ambrush | Sac-spawner | Mesopelagic | Amyelinated |
| *Cosmocalanus darwini* | 1.8 | Carnivore | Ambrush | Sac-spawner | Mesopelagic | Myelinated |
| *Ctenocalanus vanus* | 2.58 | Omnivore, Herbivore | Filter | Broadcaster | Epipelagic | Myelinated |
| *Disseta palumbii* | 1.7 | Omnivore, Herbivore | Filter | Broadcaster | Epipelagic | Amyelinated |
| *Euchaeta marina* | 8.3 | Carnivore | Filter | Sac-spawner | Mesopelagic | Myelinated |
| *Euaugaptilus facilis* | 3.90 | Carnivore | Mixed | Sac-spawner | Mesopelagic | Amyelinated |
| *Euaugaptilus hecticus* | 5.91 | Omnivore, Carnivore | Ambrush | Broadcaster | Bathypelagic | Amyelinated |
| *Euaugaptilus magnus* | 2.85 | Omnivore, Carnivore | Ambrush | Broadcaster | Mesopelagic | Amyelinated |
| *Euaugaptilus palumbii* | 8.90 | Omnivore, Carnivore | Ambrush | Broadcaster | Bathypelagic | Amyelinated |
| *Eucalanus attenuatus* | 2.70 | Omnivore, Carnivore | Ambrush | Broadcaster | Bathypelagic | Myelinated |
| *Eucalanus hyalinus* | 7.30 | Omnivore, Herbivore | Filter | Broadcaster | Mesopelagic | Myelinated |
| *Euchaeta acuta* | 8.25 | Omnivore | Filter | Broadcaster | Mesopelagic | Myelinated |
| *Euchaeta media* | 4.7 | Carnivore | Filter | Sac-spawner | Mesopelagic | Myelinated |
| *Euchaeta spinosa* | 4.82 | Carnivore | Filter | Sac-spawner | Bathypelagic | Myelinated |
| *Euchirella amoena* | 7.21 | Carnivore | Filter | Sac-spawner | Bathypelagic | Myelinated |
| *Euchirella bitumida* | 4.00 | Omnivore, Detritivore | Mixed | Sac-spawner | Bathypelagic | Myelinated |
| *Euchirella curticauda* | 7.10 | Omnivore, Detritivore | Mixed | Sac-spawner | Bathypelagic | Myelinated |
| *Euchirella maxima* | 4.94 | Omnivore, Detritivore | Mixed | Sac-spawner | Bathypelagic | Myelinated |
| *Euchirella messinensis* | 8.70 | Omnivore, Detritivore | Mixed | Sac-spawner | Bathypelagic | Myelinated |
| *Euchirella pulchra* | 6.2 | Omnivore, Detritivore | Mixed | Sac-spawner | Bathypelagic | Myelinated |
| *Euchirella rostrata* | 4.40 | Omnivore, Detritivore | Mixed | Sac-spawner | Bathypelagic | Myelinated |
| *Euchirella spinosa* | 4.07 | Omnivore, Detritivore | Mixed | Sac-spawner | Bathypelagic | Myelinated |
| *Euchirella splendens* | 6.20 | Omnivore, Detritivore | Mixed | Sac-spawner | Bathypelagic | Myelinated |
| *Euchirella truncata* | 5.20 | Omnivore, Detritivore | Mixed | Sac-spawner | Bathypelagic | Myelinated |
| *Farranula carinata* | 6.83 | Omnivore, Detritivore | Mixed | Sac-spawner | Bathypelagic | Amyelinated |
| *Farranula gracilis* | 0.98 | Carnivore | Cruise | Sac-spawner | Epipelagic | Amyelinated |
| *Gaetanus armiger* | 1.10 | Carnivore | Ambrush | Sac-spawner | Epipelagic | Myelinated |
| *Gaetanus brevicornis* | 4.70 | Omnivore | Cruise | Broadcaster | Bathypelagic | Myelinated |
| *Gaetanus kruppii* | 5.40 | Omnivore, Herbivore | Filter | Broadcaster | Bathypelagic | Myelinated |
| *Gaetanus miles* | 5.70 | Omnivore | Cruise | Broadcaster | Bathypelagic | Myelinated |
| *Gaetanus minor* | 4.70 | Omnivore | Cruise | Broadcaster | Mesopelagic | Myelinated |
| *Gaetanus pileatus* | 2.45 | Omnivore, Herbivore | Filter | Broadcaster | Bathypelagic | Myelinated |
| *Gaetanus secundus* | 6.70 | Omnivore | Cruise | Broadcaster | Bathypelagic | Myelinated |
| *Gaetanus tenuispinus* | 5.20 | Omnivore | Cruise | Broadcaster | Bathypelagic | Myelinated |
| *Gaussia prínceps* | 4.00 | Omnivore | Cruise | Broadcaster | Bathypelagic | Amyelinated |
| *Haloptilus acutifrons* | NA | NA | NA | NA | NA | NA |
| *Haloptilus angusticeps* | 4.66 | Omnivore, Carnivore | Ambrush | Broadcaster | Bathypelagic | Amyelinated |
| *Haloptilus fons* | 3.6 | Omnivore, Carnivore | Ambrush | Broadcaster | Bathypelagic | Amyelinated |
| *Haloptilus longicornis* | NA | NA | NA | NA | NA | NA |
| *Haloptilus oxycephalus* | 2.63 | Omnivore, Carnivore | Ambrush | Broadcaster | Bathypelagic | Amyelinated |
| *Haloptilus plumosus* | 5.2 | Omnivore, Carnivore | Ambrush | Broadcaster | Bathypelagic | Amyelinated |
| *Haloptilus spiniceps* | 4.20 | Omnivore, Carnivore | Ambrush | Broadcaster | Bathypelagic | Amyelinated |
| *Heterorhabdus abyssalis* | 5.45 | Omnivore, Carnivore | Cruise | Broadcaster | Mesopelagic | Amyelinated |
| *Heterorhabdus austrinus* | 3.73 | Omnivore, Carnivore | Filter | Broadcaster | Bathypelagic | Amyelinated |
| *Heterorhabdus clausii* | 4.05 | Omnivore, Carnivore | Filter | Broadcaster | Bathypelagic | Amyelinated |
| *Heterorhabdus lobatus* | 2.65 | Carnivore | Cruise | Broadcaster | Bathypelagic | Amyelinated |
| *Heterorhabdus norvegicus* | 2.64 | Omnivore, Carnivore | Filter | Broadcaster | Mesopelagic | Amyelinated |
| *Heterorhabdus oikomenikus* | 4.60 | Omnivore, Carnivore | Filter | Broadcaster | Bathypelagic | Amyelinated |
| *Heterorhabdus papilliger* | 2.66 | Omnivore, Carnivore | Filter | Broadcaster | Bathypelagic | Amyelinated |
| *Heterorhabdus spinifer* | 1.90 | Omnivore, Carnivore | Filter | Broadcaster | Bathypelagic | Amyelinated |
| *Heterorhabdus spinifrons* | 4 | Omnivore, Carnivore | Filter | Broadcaster | Bathypelagic | Amyelinated |
| *Labidocera acutifrons* | 4.70 | Omnivore | Filter | Broadcaster | Epipelagic | Amyelinated |
| *Labidocera neri* | 3.70 | Omnivore | Filter | Broadcaster | Epipelagic | Amyelinated |
| *Lophothrix frontalis* | NA | NA | NA | NA | NA | NA |
| *Lophothrix humilifrons* | NA | NA | NA | NA | NA | NA |
| *Lophothrix latipes* | NA | NA | NA | NA | NA | NA |
| *Lubbockia aculeata* | 2.84 | Omnivore | Ambrush | Sac-spawner | Mesopelagic | Amyelinated |
| *Lubbockia squillimana* | 2 | Omnivore | Ambrush | Sac-spawner | Mesopelagic | Amyelinated |
| *Lucicutia bicornuta* | 8.40 | Omnivore, Herbivore | Filter | Broadcaster | Bathypelagic | Amyelinated |
| *Lucicutia clausi* | 2.16 | Omnivore | Filter | Broadcaster | Mesopelagic | Amyelinated |
| *Lucicutia curta* | 2.90 | Omnivore, Herbivore | Filter | Broadcaster | Bathypelagic | Amyelinated |
| *Lucicutia flavicornis* | 2.5 | Omnivore | Filter | Broadcaster | Bathypelagic | Amyelinated |
| *Lucicutia gaussae* | 1.6 | Omnivore | Filter | Broadcaster | Mesopelagic | Amyelinated |
| *Lucicutia gemina* | 1.9 | Omnivore | Filter | Broadcaster | Mesopelagic | Amyelinated |
| *Lucicutia grandis* | 6.50 | Omnivore, Herbivore | Filter | Broadcaster | Bathypelagic | Amyelinated |
| *Lucicutia longicornis* | 2.00 | Omnivore, Herbivore | Filter | Broadcaster | Bathypelagic | Amyelinated |
| *Lucicutia longiserrata* | 3.00 | Omnivore, Herbivore | Filter | Broadcaster | Bathypelagic | Amyelinated |
| *Lucicutia magna* | 3.90 | Omnivore, Herbivore | Filter | Broadcaster | Bathypelagic | Amyelinated |
| *Lucicutia maxima* | 9.30 | Omnivore, Herbivore | Filter | Broadcaster | Bathypelagic | Amyelinated |
| *Lucicutia ovalis* | 2 | Omnivore | Filter | Broadcaster | Mesopelagic | Amyelinated |
| *Mecynocera clausi* | 1.29 | Omnivore, Herbivore | Filter | Broadcaster | Epipelagic | Myelinated |
| *Megacalanus princeps* | NA | NA | NA | NA | NA | NA |
| *Mesocalanus tenuicornis* | 3.4 | Omnivore, Herbivore | Filter | Broadcaster | Bathypelagic | Myelinated |
| *Metridia brevicauda* | 2.25 | Omnivore | Cruise | Broadcaster | Bathypelagic | Amyelinated |
| *Metridia curticauda* | 3.80 | Omnivore | Cruise | Broadcaster | Bathypelagic | Amyelinated |
| *Metridia longa* | 4.50 | Omnivore | Cruise | Broadcaster | Bathypelagic | Amyelinated |
| *Metridia lucens* | 4.00 | Omnivore | Cruise | Broadcaster | Bathypelagic | Amyelinated |
| *Metridia princeps* | 9.00 | Omnivore | Cruise | Broadcaster | Bathypelagic | Amyelinated |
| *Metridia venusta* | 3.15 | Omnivore | Cruise | Broadcaster | Bathypelagic | Amyelinated |
| *Miracia efferata* | NA | NA | NA | NA | NA | NA |
| *Nannocalanus minor* | 2.45 | Omnivore, Herbivore | Filter | Broadcaster | Mesopelagic | Myelinated |
| *Neocalanus gracilis* | 4.4 | Omnivore, Herbivore | Filter | Broadcaster | Mesopelagic | Myelinated |
| *Neocalanus robustior* | 4.65 | Omnivore, Herbivore | Filter | Broadcaster | Bathypelagic | Myelinated |
| *Nullosetigera aequalis* | NA | NA | NA | NA | NA | NA |
| *Nullosetigera bidentata* | NA | NA | NA | NA | NA | NA |
| *Nullosetigera helgae* | NA | NA | NA | NA | NA | NA |
| *Nullosetigera impar* | NA | NA | NA | NA | NA | NA |
| *Oithona atlantica* | 1.43 | Omnivore | Ambrush | Sac-spawner | Mesopelagic | Amyelinated |
| *Oithona plumifera* | 1.54 | Omnivore | Ambrush | Sac-spawner | Mesopelagic | Amyelinated |
| *Oithona robusta* | 1.65 | Omnivore | Ambrush | Sac-spawner | Epipelagic | Amyelinated |
| *Oithona setigera* | 2.04 | Omnivore | Ambrush | Sac-spawner | Mesopelagic | Amyelinated |
| *Oithona tenuis* | 1.37 | Omnivore | Ambrush | Sac-spawner | Epipelagic | Amyelinated |
| *Oncaea conifera* | 1.50 | Omnivore, Detritivore | Cruise | Sac-spawner | Mesopelagic | Amyelinated |
| *Oncaea media* | 1.02 | Omnivore, Detritivore | Cruise | Sac-spawner | Mesopelagic | Amyelinated |
| *Oncaea mediterranea* | 1.60 | Omnivore, Detritivore | Cruise | Sac-spawner | Mesopelagic | Amyelinated |
| *Oncaea venusta* | 1.7 | Omnivore, Detritivore | Cruise | Sac-spawner | Mesopelagic | Amyelinated |
| *Pachos punctatum* | NA | NA | NA | NA | NA | NA |
| *Paracalanus aculeatus* | 1.45 | Omnivore, Herbivore | Filter | Broadcaster | Epipelagic | Myelinated |
| *Paracalanus denudatus* | 0.96 | Omnivore, Herbivore | Filter | Broadcaster | Epipelagic | Myelinated |
| *Paracalanus indicus* | 1.30 | Omnivore, Herbivore | Filter | Broadcaster | Epipelagic | Myelinated |
| *Paracalanus nanus* | 0.65 | Omnivore, Herbivore | Filter | Broadcaster | Mesopelagic | Myelinated |
| *Paracalanus parvus* | 1.3 | Omnivore, Herbivore | Filter | Broadcaster | Epipelagic | Myelinated |
| *Paraeuchaeta exigua* | 7.66 | Carnivore | Mixed | Sac-spawner | Bathypelagic | Myelinated |
| *Paraeuchaeta gracilis* | 7.04 | Carnivore | Mixed | Sac-spawner | Bathypelagic | Myelinated |
| *Paraheterorhabdus compactus* | NA | NA | NA | NA | NA | NA |
| *Paraugaptilus buchani* | NA | NA | NA | NA | NA | NA |
| *Phaenna spinifera* | 3.02 | Carnivore | Filter | Broadcaster | Mesopelagic | Myelinated |
| *Pleuromamma abdominalis* | 4.5 | Omnivore | Filter | Broadcaster | Mesopelagic | Amyelinated |
| *Pleuromamma borealis* | 2.5 | Omnivore | Filter | Broadcaster | Mesopelagic | Amyelinated |
| *Pleuromamma gracilis* | 2.55 | Omnivore | Filter | Broadcaster | Mesopelagic | Amyelinated |
| *Pleuromamma piseki* | 2.40 | Omnivore | Filter | Broadcaster | Mesopelagic | Amyelinated |
| *Pleuromamma quadrungulata* | 5.00 | Omnivore | Filter | Broadcaster | Bathypelagic | Amyelinated |
| *Pleuromamma robusta* | 4.90 | Omnivore | Filter | Broadcaster | Bathypelagic | Amyelinated |
| *Pleuromamma xiphias* | 5.87 | Omnivore | Filter | Broadcaster | Bathypelagic | Amyelinated |
| *Pontellina plumata* | 1.94 | Carnivore | Ambrush | Broadcaster | Mesopelagic | Amyelinated |
| *Pseudhaloptilus abbreviatus* | NA | NA | NA | NA | NA | NA |
| *Pseudhaloptilus eurygnathus* | NA | NA | NA | NA | NA | NA |
| *Pseudoamallothrix emarginata* | NA | NA | NA | NA | NA | NA |
| *Pseudoamallothrix obtusifrons* | NA | NA | NA | NA | NA | NA |
| *Rhincalanus cornutus* | 4.16 | Omnivore, Herbivore | Filter | Broadcaster | Bathypelagic | Myelinated |
| *Rhincalanus gigas* | 10.00 | Omnivore, Herbivore | Filter | Broadcaster | Bathypelagic | Myelinated |
| *Rhincalanus nasutus* | 6.1 | Omnivore, Herbivore | Filter | Broadcaster | Bathypelagic | Myelinated |
| *Scaphocalanus affinis* | 5.40 | Omnivore, Detritivore | Filter | Broadcaster | Bathypelagic | Myelinated |
| *Scaphocalanus angulifrons* | 6.30 | Omnivore, Detritivore | Filter | Broadcaster | Bathypelagic | Myelinated |
| *Scaphocalanus brevicornis* | 2.66 | Omnivore, Detritivore | Filter | Broadcaster | Bathypelagic | Myelinated |
| *Scaphocalanus brevirostris* | 2.04 | Omnivore, Detritivore | Filter | Broadcaster | Bathypelagic | Myelinated |
| *Scaphocalanus curtus* | 1.76 | Omnivore, Detritivore | Filter | Broadcaster | Bathypelagic | Myelinated |
| *Scaphocalanus echinatus* | 2.56 | Omnivore, Detritivore | Filter | Broadcaster | Bathypelagic | Myelinated |
| *Scaphocalanus elongatus* | 3.20 | Omnivore, Detritivore | Filter | Broadcaster | Bathypelagic | Myelinated |
| *Scaphocalanus emine* | 3.20 | Omnivore, Detritivore | Filter | Broadcaster | Bathypelagic | Myelinated |
| *Scaphocalanus magnus* | 5.60 | Omnivore, Detritivore | Filter | Broadcaster | Bathypelagic | Myelinated |
| *Scolecithricella abyssalis* | 2.21 | Omnivore, Detritivore | Filter | Broadcaster | Mesopelagic | Myelinated |
| *Scolecithricella dentata* | 2.07 | Omnivore, Detritivore | Filter | Broadcaster | Mesopelagic | Myelinated |
| *Scolecithricella minor* | 1.70 | Omnivore, Detritivore | Filter | Broadcaster | Bathypelagic | Myelinated |
| *Scolecithricella ovata* | 2.50 | Omnivore, Detritivore | Filter | Broadcaster | Bathypelagic | Myelinated |
| *Scolecithricella vittata* | 2.00 | Omnivore, Detritivore | Filter | Broadcaster | Mesopelagic | Myelinated |
| *Scolecithrix bradyi* | 1.61 | Omnivore, Detritivore | Cruise | Broadcaster | Epipelagic | Myelinated |
| *Scolecithrix danae* | 2.52 | Omnivore, Detritivore | Cruise | Broadcaster | Mesopelagic | Myelinated |
| *Scolecitricella tenuiserrata* | 1.22 | Omnivore, Detritivore | Filter | Sac-spawner | Mesopelagic | Myelinated |
| *Scolecitrichopsis ctenopus* | 1.70 | Omnivore, Detritivore | Ambrush | Broadcaster | Epipelagic | Myelinated |
| *Scottocalanus securifrons* | NA | NA | NA | NA | NA | NA |
| *Subeucalanus crassus* | 4.6 | Omnivore, Herbivore | Filter | Broadcaster | Mesopelagic | Myelinated |
| *Subeucalanus monachus* | 2.84 | Omnivore, Herbivore | Filter | Broadcaster | Bathypelagic | Myelinated |
| *Subeucalanus pileatus* | 2.50 | Omnivore, Detritivore | Filter | Broadcaster | Mesopelagic | Myelinated |
| *Subeucalanus subtenuis* | 3.70 | Omnivore, Herbivore | Filter | Broadcaster | Mesopelagic | Myelinated |
| *Temora longicornis* | 1.66 | Omnivore | Filter | Broadcaster | Epipelagic | Amyelinated |
| *Temora stylifera* | 2.05 | Omnivore | Filter | Broadcaster | Epipelagic | Amyelinated |
| *Temoropia mayumbaensis* | NA | NA | NA | NA | NA | NA |
| *Undeuchaeta incisa* | 6.66 | Omnivore, Carnivore | Filter | Sac-spawner | Bathypelagic | Myelinated |
| *Undeuchaeta major* | 6.50 | Omnivore, Carnivore | Filter | Sac-spawner | Bathypelagic | Myelinated |
| *Undeuchaeta plumosa* | 4.65 | Omnivore, Carnivore | Filter | Sac-spawner | Mesopelagic | Myelinated |
| *Undinula vulgaris* | 3.25 | Omnivore, Herbivore | Filter | Broadcaster | Mesopelagic | Myelinated |
| *Xantocalanus agilis* | NA | NA | NA | NA | NA | NA |

**Supplementary Table 2** Description of the six functional traits selected for copepods species.

| Traits | Trait type | Ecological function | Definition | Implementation |
| --- | --- | --- | --- | --- |
| Trophic regime | Behavioural | Feeding | The primary food sources to describe the role in food-webs: omnivore-herbivore, omnivore-carnivore, omnivore-detritivore, strict carnivore, and omnivore. | Factor: omnivore-herbivore, omnivore-carnivore, omnivore-detritivore, strict carnivore, and omnivore |
| Feeding mode | Behavioural | Feeding | The predation strategy of the copepods: ambush, current, cruise and, mixed. The latter refers to species that show both feeding strategies (current-ambush and current-cruise). | Factor: ambush, current (filter), cruise and mixed |
| Spawning strategy | Life history | Growth and reproduction | Copepods were separated into two reproductive groups depending on whether they can release eggs into the waters after fertilisation (free or diffusive spawning) or whether they can be transported by females in egg sacs or egg masses (sac spawning). | Factor: broadcaster and sac-spawning |
| Myelination | Morphological | Survival | Myelinated copepods have a lipid-rich myelin sheath around their nerves that allows faster attack or avoidance reactions and is related to feeding rates and improved energy conservation at low food conditions. For this trait we assume that is conserved in the taxonomy. | Factor: amyelinated and myelinated |
| Vertical distribution | Behavioural | Feeding, and survival | The deepest layer in which they can be found, there are three layers: epipelagic, mesopelagic, and bathypelagic. | Ordered factor: epipelagic > mesopelagic > bathypelagic |
| Body size | Morphological | Feeding, survival and, growth and reproduction | The average of adult female body length (mm) | Numeric |

**Supplementary Table 3** *Sperman’s* correlation between *α*-diversity indices (taxonomic and functional). *FDis*, functional disparity; *FDiv*, functional divergence; *FEve*, fucntional evenness; *FOri*, functional originality; *FSpe*, functional specialization; *SR*, species richness; *= p< 0.05; **= p< 0.01; ***= p< 0.001; *Δ*+, average taxonomic distinctiveness; *Λ*+, variation in taxonomic distinctiveness. *= *p*< 0.05, **= *p*< 0.01 ***= *p*< 0.001.

| **Indices** | *SR* | ***Δ+*** | ***Λ+*** |
| --- | --- | --- | --- |
| *FDis* | 0.311*** | 0.570*** | 0.311*** |
| *FEve* | -0.225** | 0.322*** | -0.225** |
| *FDiv* | 0.125 | -0.050 | 0.125 |
| *FOri* | 0.141 | 0.108 | 0.141 |
| *FSpe* | 0.313*** | 0.479*** | 0.313*** |

**Supplementary Table 4** *Sperman*’s correlation between environmental variables. *= *p*< 0.05, **= *p*< 0.01 ***= *p*< 0.001.

| **Explanatory variables** | ***Cond*** | ***F*** | ***Tur*** | ***T*** | ***D*** | ***S*** | ***O_2_*** | |  |
| --- | --- | --- | --- | --- | --- | --- | --- | --- | --- |
| *Conductivity (Cond)* | - |  |  |  |  |  | |  | |
| *Fluorescence (F)* | 0.590*** | - |  |  |  |  | |  | |
| *Turbulence (Tur)* | 0.582*** | 0.911*** | - |  |  |  | |  | |
| *Temperature (T)* | 0.999*** | 0.603*** | 0.590*** | - |  |  | |  | |
| *Density (D)* | -0.944*** | -0.744*** | -0.692*** | -0.952*** | - |  | |  | |
| *Salinity (S)* | 0.960*** | 0.452*** | 0.479*** | 0.953*** | -0.832*** | - | |  | |
| *Oxygen (O_2_)* | 0.445*** | 0.649*** | 0.626*** | 0.449*** | -0.506*** | 0.369*** | | - | |
| *Chlorophyll (Chl)* | -0.175* | 0.189 | 0.133 | -0.167 | 0.046* | -0.241*** | | 0.114 | |
